# 

**Published:** 1990-09

**Authors:** 


W1 WE403

V.105 NO.3 1990

C.01--------SEQ: SR0068788

TI: WEST OF ENGLAND MEDICAL

JOURNAL

ND
9

### WEST OF ENGLAND MEDICAL JOURNAL

**Volume 105(iii)** **September 1990**

**The Ups and Downs of a Medical Referee**
**Penile Rods – the Southmead Experience**
**MRI in Alzheimer's Disease**
**Screening for Cervical Cancer**
**Children of the Nineties**
**Explosion in the size of the Elderly Population**
**Management of Lithium Toxicity**
**HIV in Zaire**

**FROM OUR CORRESPONDENTS**
**REPORTS OF MEETINGS ETC**

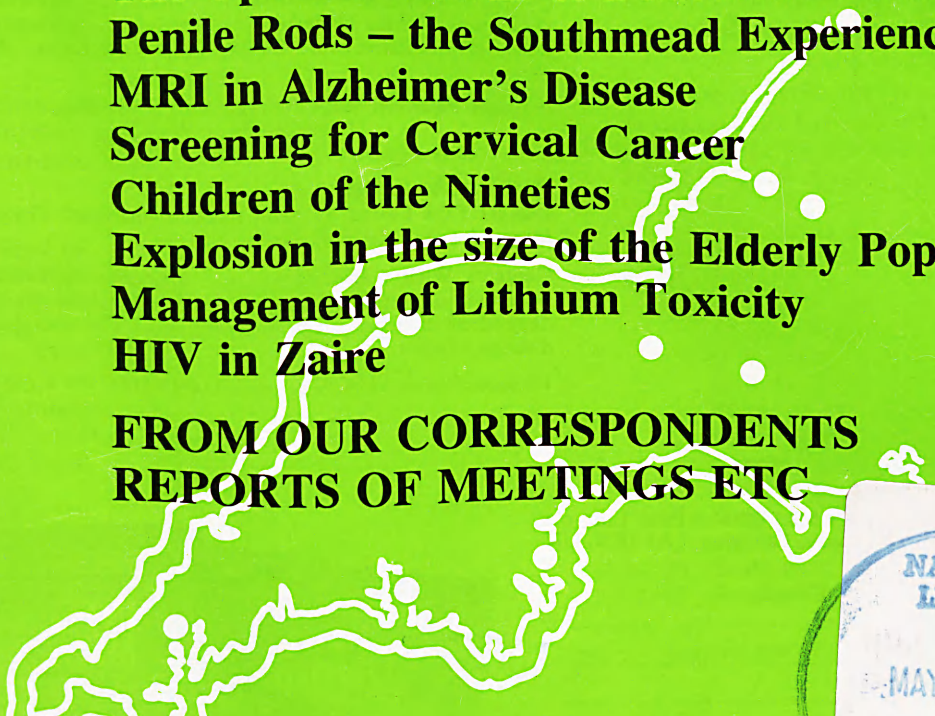

NATIONAL LIBRARY
MAY 02 1991
OF MEDICINE

FORMERLY BRISTOL MEDICO-CHIRURGICAL JOURNAL

ISSN: 0960 6440 NLM Code B5Q

